# Buffalo General Hospital

**Published:** 1909-03

**Authors:** 


### BUFFALO MEDICAL JOURNAL.

**A Monthly Review of Medicine and Surgery.**

*EDITOR:*

WILLIAM WARREN POTTER, M. D.

All communications, whether of a literary or business nature, books for reviewand exchanges, should be addressed to the editor, 238 DELAWARE AVENUE, BUFFALO, N. Y,

VOL. LXIV. MARCH, 1909. No. 8

### Buffalo General Hospital.

THIS excellent institution for the care of the sick or injured recently celebrated its golden anniversary. It was opened for the reception of patients in November, 1858, in a building that constitutes a wing of the present structure. The hospital building proper has grown so that it now nearly or quite quadruples the original structure in size. Besides, it has many or several additional buildings that belong to the hospital—a mortuary, a nurse's home, a child's hospital, and other minor houses. Taken in its entirety it may be said with a truth that Buffalo General Hospital is one of the better institutions of its class in America. Its fiftieth annual report deserves careful examination.

The *News* (Buffalo), in its Sunday edition of February 14, 1909, made the hospital the subject of its leading editorial. We take pleasure in reprinting because it contains much that should appeal to every public spirited citizen in Buffalo and vicinity. And this brings us to say that the surrounding cities and towns, —Niagara Falls, Lockport, Jamestown, Dunkirk, Tonawanda,— are all interested, or should be in the prosperity of the hospital. We will now let the *News* speak.

HELP THE GENERAL HOSPITAL.

In another column is printed a resume of the fiftieth annual report of the Buffalo General Hospital, which tells of the vast amount of good done in the past year and shows the usual financial deficit which seems to be the inevitable reward of large general hospital service. The Buffalo General Hospital has grown to be one of the finest institutions of its kind in this country. It is supplied with the latest and most perfect equipment for the treatment of diseases, both surgical and medical; its medical and surgical staffs are headed by men whose reputations are not purely local, men whose opinions are accepted as authoritative in both this country and Europe; every branch of medicine and surgery is supplied by able workers whose every need in the fields of their special activity in the way of apparatus and appliances is at their command.

The hospital proper has been stripped as much as possible of the usual institutional atmosphere, and the wards, private rooms, buildings and grounds have been beautified; there are arbors, balconies, pergolas and sun parlors and a roof garden all inclosed in glass. All this has been done not without great effort on the part of the trustees, and it has been done without expense to the funds which are devoted to the maintenance of the hospital and to the care of the patients. All the asthetic details of beautifying the buildings and grounds have either been donated by friends of the institution or by the trustees as individuals and as a board.

An institution such as the Buffalo General Hospital cannot be conducted as a purely commercial enterprise. So long as there is not sufficient money forthcoming for its maintenance there must be deficits year after year which can only be wiped out eventually by donations and legacies. Of the donations there are unfortunately too few. The endowment and the legacy are what count for results, permanent in character, in the maintenance of great charities, and the president of the General Hospital's board of trustees makes a suggestion which, if followed by the men and women of wealth, would place the General Hospital in a comparatively few years on a safe, self-supporting basis:

"Why should we not follow up our good intentions and attend now to our wills and so make the great Buffalo General Hospital the pride of charity-loving people."

That is far from a begging appeal for funds. It is a practical suggestion which should be taken to heart. No matter how small the legacy it will help in the splendid work which the hospital is doing and give it opportunity for more extensive work for the good of the people who are in the grip of disease. Each year that the city grows there is increased demand for hospital facilities, and even with the new wings and new buildings which have been added in recent years the hospital is often crowded. To meet the requirements of increasing population the support of the people is needed.

"Attend now to our wills" and the Buffalo General Hospital will become one of the greatest, as it is now one of the best conducted and most beautiful hospitals in the United States.

---

PRESIDENT Fallières's first list of decorations for the New Year (*The Tribune*), included the name of one man who is little known. This was a young physician named Louis Bozy, who lost an eye in the discharge of his duty. Dr. Bozy, while acting as assistant to an operating surgeon in one of the Paris hospitals, had an eye injured through a drop of poisonous matter coming in contact with it. He knew that an antidote must be applied immediately, but by doing this he would have left the chief surgeon unattended, and remained at his post with his eye uncared for until the operation was completed. In consequence of his heroism he lost an eye and was confined to a hospital for a long time. President Fallières, in conferring the decoration, said that a wound received by a physician in the discharge of his duty was as honorable as one received on the battlefield.

---

THE pure milk crusade of the Chicago Health Department now is about to encroach on the hitherto exclusive preserves of the saloon, soda water stand and other public thirst-quenching establishments. Dr. W. A. Evans, Health Commissioner, believes that when the milk is sufficiently purified so that it doesn't contain the germs of everything from stomachache to typhoid fever, the public should have opportunity to drop in and drink it, at every convenient corner. "He has gathered statistics and reports quietly, and the latest, which he has had translated at considerable labor, described the public milk depots of Buenos Ayres, where more than a million glasses of milk are sold daily. In the Argentine Republic milk almost has become the national tipple. Such a situation, Dr. Evans believes, would not be bad for this country," says *The Chicago News*.

---

"IF London were painted green," says a leading eye specialist, "it would have a wonderful effect upon the health and spirits of the Londoners." Hitherto all efforts to improve health and spirits by changing the color scheme of a city have taken the direction of painting the town red.

---

THE need for a hospital for contagious diseases has been shown again.—*Express*.

Yes, and if it takes our so-called city government relatively as long to build it as it has done to clear our streets of ice and snow after one sleet storm, we may await in patience the coming of the millennium.

